# A self-powered microsystem with efficient power management for continuous wireless sensing

**DOI:** 10.1038/s41378-026-01315-z

**Published:** 2026-05-13

**Authors:** Xiangyu Zhao, Zerui Xu, Ziyang Ou, Qingfeng Wu, Yifeng Liu, Yuqi Kang, Philippe Basset, Xiaohong Wang

**Affiliations:** 1 School of Integrated Circuits, Tsinghua University, Beijing, China; 2 Univ Gustave Eiffel, CNRS, ESYCOM, Marne-la-Vallée, France

**Keywords:** Engineering, Chemistry

## Abstract

The rapid expansion of the Internet of Things (IoT) has increased the demand for self-powered wireless microsystems, with energy harvesters emerging as a key approach for powering distributed IoT nodes. Among energy harvesters, triboelectric nanogenerators (TENGs) are promising for harvesting mechanical energy; yet converting their high-voltage, low-current pulsed output into a stable, low-voltage supply for microsystems remains a key challenge. This work introduces a self-powered microsystem capable of continuous sensing and wireless communication, powered exclusively by a TENG harvesting low-frequency mechanical energy. System-level integration and optimization address the impedance mismatch between the energy harvester and the microsystem electronics, enabling a five-fold increase in harvested energy compared to conventional full-bridge rectification. The system cold-starts from 0 V to 4.2 V within 525 s and transitions to high-efficiency power management, providing ~110 μW under 5 Hz mechanical excitation. Enabled by low-power system operation, a self-powered wireless gas monitoring system is demonstrated. This system establishes a pathway toward battery-less IoT nodes and highlights the potential of TENG-powered microsystems for long-term, maintenance-free IoT applications.

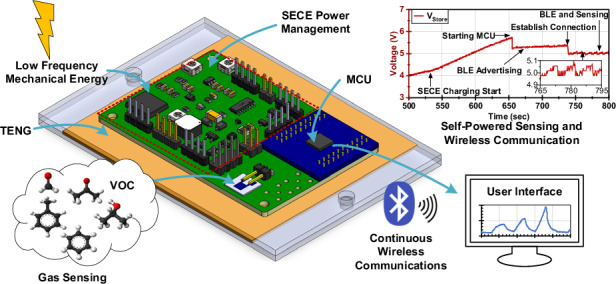

## Introduction

As the Internet of Things (IoT) continues to expand, reliable power delivery to distributed wireless systems capable of continuous sensing and wireless communication is critical for applications such as personal monitoring, smart cities, industrial monitoring, and environmental monitoring^1–6^. However, powering these systems remains a bottleneck, as frequent recharging or replacement of the traditional batteries used is impractical due to short lifespans, high costs, and environmental impact^7–9^. These challenges have caused intensive interest in self-powered microsystems that harvest ambient energy to sustain system operation, which is also a key enabler for emerging embodied intelligence in distributed physical systems^10–12^.

Mechanical energy is particularly attractive because motion is ubiquitous^12,13^ and much of this energy exists in the low-frequency range (<10 Hz), such as human motion, ocean waves, and structural vibrations^13–18^. Low-frequency operation poses challenges for resonant electromagnetic and piezoelectric energy harvesters, whose output drops sharply outside narrow resonant bands^13^. On the other hand, triboelectric nanogenerators (TENGs) generate charge through repeated contact–separation or sliding motions, and unlike resonance-based energy harvesters, they do not rely on narrowband resonance for efficient operation and can maintain effective energy conversion performance even under irregular, low-frequency, or intermittent mechanical excitations typical of human motion and environmental vibrations^16,17^. Combined with their high output, material versatility, and low cost, TENGs have attracted significant research interest, with a focus on the development of novel materials, surface modifications, and structural optimizations to maximize charge generation and power density^15,17,19^.

However, the efficient transfer of energy generated by TENGs remains a major challenge. Their characteristic high-voltage (often several hundreds of volts), low-current pulsed outputs (μA-level), and pF-level inner capacitance cause severe mismatches when interfacing with conventional low-voltage IoT electronics^19–21^. These mismatches result in substantial energy loss and low power transfer efficiency, limiting the capacity of TENGs to sustain the high power requirements of microsystem operation, particularly for applications that require continuous sensing and wireless communication. As a result, TENGs have most commonly been leveraged to function as self-powered sensors, where the mechanical stimulus encodes sensing information, rather than serving as power sources^22,23^.

To overcome these limitations, prior research has relied on higher frequency mechanical sources, multiple TENGs, and hybrid energy harvesters to meet the power requirements of microsystems. High-frequency energy sources, such as wind^24,25^ or raindrops^26^, can generate significant energy. However, their applicability is limited, making them unsuitable for applications dominated by low-frequency motion. Multiple TENGs have been combined in parallel to increase power for self-powered microsystems with wireless capabilities^27,28^, but the large number of devices needed often hinders miniaturization. Another promising path of investigation is the development of hybrid energy harvesters, where TENGs are combined with other types of energy harvesters, such as electromagnetic^29,30^, piezoelectric^31^, thermal^32^, or biochemical^33^. While these hybrid solutions achieve higher power generation and better energy use, the TENG typically contributes less than its counterpart, and the added complexity, cost, and size often make the approach unjustified. Significant research has also focused on power management circuits to improve TENG energy extraction^20,21,34,35^. However, these studies often focus solely on the TENG and the power management circuits, rarely demonstrating performance at the full system level. To the best of our knowledge, continuous, self-powered wireless operation using a single TENG harvesting low-frequency mechanical energy has not yet been demonstrated.

This paper presents a complete microsystem that integrates a TENG energy harvester, a high-voltage-tolerant power management circuit with cold-start capability, a microfabricated gas sensor, and a microcontroller for continuous operation powered solely by a single low-frequency TENG. The cold-start circuit enables reliable system initialization from a fully uncharged state using only TENG-harvested energy, eliminating the need for external or pre-charged energy sources. By efficiently converting the TENG’s pulsed output into a stable low-voltage supply with a power management circuit, sufficient energy to power continuous operation from low-frequency motion is obtained. To our knowledge, this system represents the first demonstration of a wireless sensing system operating continuously while powered solely by a single TENG harvesting low-frequency mechanical energy, thereby advancing the feasibility of self-powered systems for continuous, real-time monitoring in wearable, environmental, and industrial applications.

## Results and Discussion

### Microsystem architecture and design

**Fig. 1:**
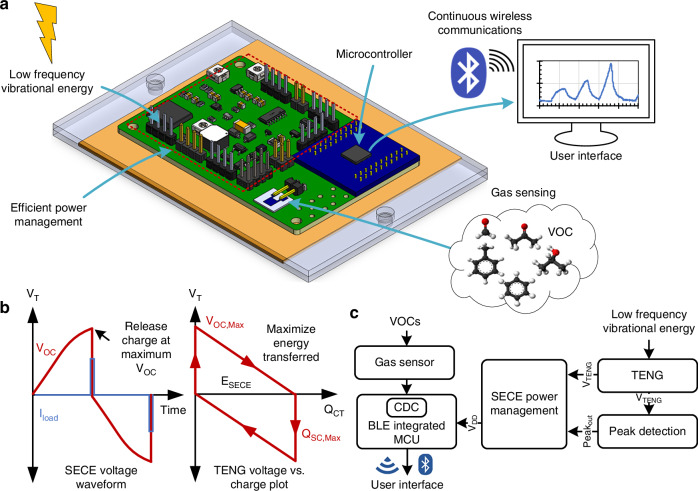
Self-powered microsystem supporting continuous wireless sensing. **a** Three-dimensional illustration of the self-powered microsystem that harvests low-frequency mechanical energy for continuous wireless sensing and communication. **b** The operating principle of SECE power management. **c** Simplified block diagram of the self-powered microsystem

The self-powered microsystem in this work is designed to monitor exposure to chemical vapors, consisting of three main components: the energy harvester, the power management circuit, and the sensor and microcontroller unit (MCU) (Fig. 1a). The energy harvester is a 10 cm by 10 cm contact-separation mode TENG. It utilizes polytetrafluoroethylene (PTFE) as the electronegative layer and polyimide (PI) as the electropositive layer, a material pairing selected for their significant triboelectric potential difference and simplicity of fabrication. To ensure repeatable contact-separation motion and minimize wear, the TENG is mounted using two sets of linear bearings and springs, which provide compliant mechanical support and consistent actuation behavior. The TENG is actuated using periodic mechanical vibration at 2-5 Hz to simulate the low-frequency human-motion-induced mechanical movement.

A highly integrated PCB, measuring 4 cm by 7 cm, is responsible for handling the pulsed TENG output with a power management circuit, a gas sensor readout, and a low-power MCU for processing and continuously transmitting sensor data. The power management circuit is designed based on a synchronous electric charge extraction (SECE) strategy, which allows the TENG to remain in an open-circuit state until its output voltage peaks (Fig. 1b). At this point, the stored charge is rapidly extracted, significantly improving energy transfer efficiency compared to other power management methods, as it allows a higher voltage to be reached^20^. The SECE power management circuit includes a peak detection circuit, a cold-start circuit, a flyback transformer, and a monostable multivibrator. These components are designed to operate at high voltages of up to 600 V generated by the TENG.

The sensing element is a capacitive-type volatile organic compound (VOC) sensor microfabricated using interdigitated electrodes (7 μm width and gap and active area of 5 mm^2^), coated with a thin polydimethylsiloxane (PDMS) layer that serves as the gas-sensitive film. Exposure to VOCs alters the dielectric constant and thickness of the PDMS, leading to measurable capacitance changes. This sensor operates at room temperature, requires no consumables, and consumes no power itself, with only the readout circuitry drawing ultra-low power. Selectivity and sensitivity can be tuned by modifying the PDMS film thickness, switching to a different sensing material, or incorporating additional functional groups^36^. The ambient VOC information from the gas sensor is then wirelessly transmitted through BLE to a graphical user interface (GUI), where the transmitted data is parsed and can either be plotted in real time or saved for further analysis.

The TENG first charges a storage capacitor directly through a cold-start circuit to allow the system to start operation in a discharged state. Once the capacitor reaches a predefined threshold of 4.2 V, the SECE power management begins operation, and the charging path provided by the cold-start circuit is switched off. The MCU performs data acquisition and transmits sensor readings via Bluetooth Low Energy (BLE). For sustained operation, the energy generated by the TENG must match or exceed the consumption of the power management, sensor, and communication modules, which is on the order of 100 μW. A simplified block diagram of the different components that make up the microsystem can be seen in Fig. 1c.

### Energy harvester and power management

#### Energy harvester characterization

**Fig. 2:**
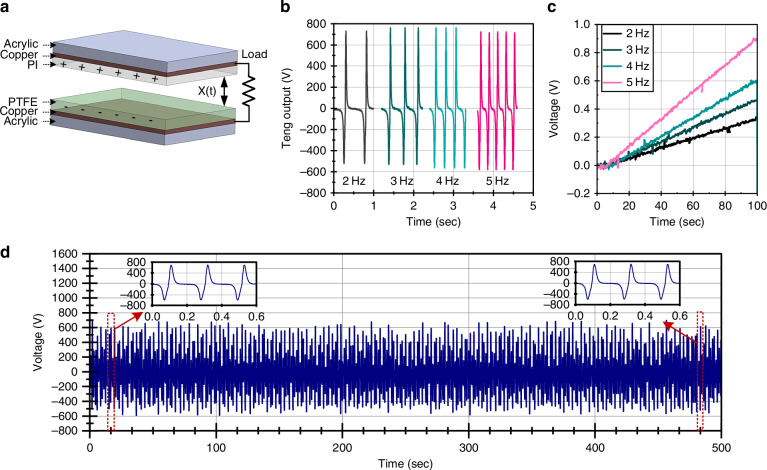
Characterization of TENG for low-frequency mechanical energy harvesting. **a** Schematic illustration of the TENG. **b** TENG operation at different frequencies. **c** Comparison of direct charging of a 220 μF capacitor by the TENG at different frequencies. **d** Stability of the TENG operation over 2500 cycles

To power the microsystem, a contact-separation mode TENG with dimensions of 10 cm × 10 cm was utilized. The device employed PTFE as the electronegative layer and polyimide as the electropositive layer, a material pairing selected for its large triboelectric potential difference and ease of fabrication. Copper served as the conductive electrode to collect and transfer the induced charges, while an acrylic backing provided structural support (Fig. 2a).

The operating principle of the TENG is based on periodic contact and separation of the two dielectric surfaces under mechanical excitation^37^, and additional details can be found in Section S1 of the Supporting Information. Upon contact, triboelectric charges of opposite polarity accumulate on each surface due to electron transfer. Subsequent separation generates an electric potential difference, inducing a displacement current through an external circuit and enabling the conversion of mechanical energy into electrical energy. Although the output and miniaturization of TENGs can be substantially improved by modifications that augment surface charge density, such as physical texturing^38–40^ for a larger effective area or active charge excitation^41,42^ for pre-charging, this work forgoes such modifications to maintain a simple, low-cost fabrication process amenable to microsystem integration.

TENG characterization was performed using a linear motor to apply periodic mechanical excitation at 2–5 Hz with a displacement of 1 cm, simulating low-frequency human motion^29^. The *C*_*max*_ of the fabricated TENG was measured to be approximately 1 nF using an impedance meter. As shown in Fig. 2b, a consistent peak-to-peak voltage of 1200 V and a maximum voltage of 720 V were maintained across this frequency range, indicating reliable contact and separation in each cycle. To evaluate the energy harvesting capability, the TENG output was connected to a 220 μF commercial capacitor through a full-wave bridge rectifier (Fig. 2c). The capacitor was selected to match the storage requirements of the microsystem, allowing sufficient voltage buildup for regulated startup while minimizing losses. At 2 Hz, the capacitor voltage increased steadily, reaching 0.29 V after 100 s, and increased further to 0.86 V at 5 Hz. The average charge transferred per cycle ranged from 300 to 400 nC, with values of 320 nC and 376 nC recorded at 2 Hz and 5 Hz, respectively.

Based on these results and the frequency range of typical human motion^43–45^, 5 Hz was selected as the standard operating point for subsequent testing, emulating the frequencies associated with vigorous limb movements or running. While periodic excitation was used for controlled characterization, real-world biomechanical motion is inherently irregular, with time-varying frequency and amplitude. The contact-separation TENG and the peak-detection-based SECE power management do not rely on strict periodicity and can operate under intermittent excitation seen in human motion. The selected displacement amplitude is comparable to typical human-motion-induced mechanical displacements (on the order of millimeters to centimeters), supporting the representativeness of the experimental conditions.

The TENG was actuated at 5 Hz for over 2500 cycles, during which the open-circuit voltage remained stable, demonstrating consistent output (Fig. 2d). The materials employed (PTFE and PI) are widely recognized for their high mechanical robustness and low wear under repeated contact-separation operation^46^. The implementation of unpatterned films in this design further reduces localized stress concentrations that can accelerate material degradation in microstructured surfaces. In this work, the demonstrated stable operation of 2500 cycles serves to validate the initial reliability of the integrated system. This performance is consistent with established PTFE-based contact-separation triboelectric systems that have reported sustained performance over extended cycling on the order of 10⁵ cycles under similar contact-separation operation^47,48^.

Successful capacitor charging confirms the TENG’s suitability for powering self-sustaining microsystems. However, while the TENG alone achieves only modest voltage rise across a large storage capacitor, the use of SECE power management is needed to improve energy transfer efficiency. The SECE power management circuit is tailored to match the TENG’s output by selecting and tuning key design parameters, including the switch pulse width, peak detection thresholds, and coupled-inductor turns ratio, thereby enhancing energy transfer efficiency while reducing losses associated with the TENG’s high internal impedance and low charge density.

#### Power management design

In order to solve the aforementioned efficiency loss during energy transfer, the high-voltage, pulsed output of TENGs requires dedicated power management. The SECE power management strategy is used to interface the TENG with the sensing and wireless circuits. In SECE, the TENG is left open-circuited during most of each cycle to allow its voltage to rise to the peak open-circuit value. When the peak voltage is reached, the TENG is connected to an inductor that rapidly draws the accumulated charge, which is then transferred to a storage capacitor. SECE decouples overall energy-extraction efficiency from the output voltage, enabling near-maximum transfer each cycle. Compared with commonly used full- and half-wave bridge rectifiers (F/H-BRs), which typically harvest only a small fraction of the TENG’s available energy^28,49,50^ without additional regulation stages^51^, SECE improves microsystem-level efficiency. Alternative approaches, such as Synchronized Switch Harvesting on Inductors (SSHI), can also enhance energy extraction^34,35,50,52^ but are sensitive to parasitic effects and operating conditions, limiting their practicality in TENG-powered microsystems^20,35,50^. Further comparison of power management strategies is provided in Section S2 of the Supporting Information.

**Fig. 3:**
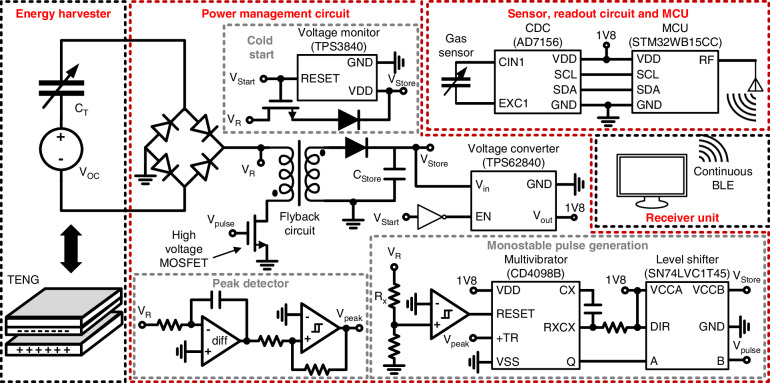
Schematic diagram of the microsystem electronics. The microsystem consists of the energy harvester, power management circuits, gas sensor, readout circuits, microcontroller (MCU), and receiver unit

Building upon our prior work on power management for TENG^20,21^, a flyback-based SECE topology is implemented to transfer the extracted energy to a low-voltage storage capacitor. The SECE power management circuit and the other microsystem electronics are shown in Fig. 3. A derivative-based peak detection circuit identifies the TENG voltage maxima, with comparator thresholds and reference voltage tuned to mitigate RC delays, parasitic effects, and noise, ensuring robust operation under low-frequency and aperiodic excitation. The detected peak triggers a monostable pulse, which controls a high-voltage switch in the flyback circuit. The switch is a GaN HEMT switch with a 600 V rating to minimize conduction and switching losses while safely handling the TENG’s high-voltage output. When the switch closes, energy from the TENG is temporarily stored in the flyback’s coupled inductor and is then released to the storage capacitor, providing voltage conversion and galvanic isolation. The energy stored in the capacitor then powers downstream sensing, processing, and wireless communication circuits. Because SECE requires an initial operating voltage, a cold-start circuit is included. During startup, the TENG charges the storage capacitor directly until sufficient energy is available to power the regulated SECE circuitry, after which the system transitions automatically to full SECE operation, allowing sustained, battery-free microsystem functionality. To minimize power consumption, SECE and other microsystem electronics operate from a 1.8 V supply. Further details of the SECE architecture and circuit optimization are provided in Section S3 of the Supporting Information, and the circuit schematic and specifications of key components (e.g., inductor, switching devices) used in the circuit implementation are summarized in Section S4 of the Supporting Information.

#### Energy harvester and power management performance

**Fig. 4:**
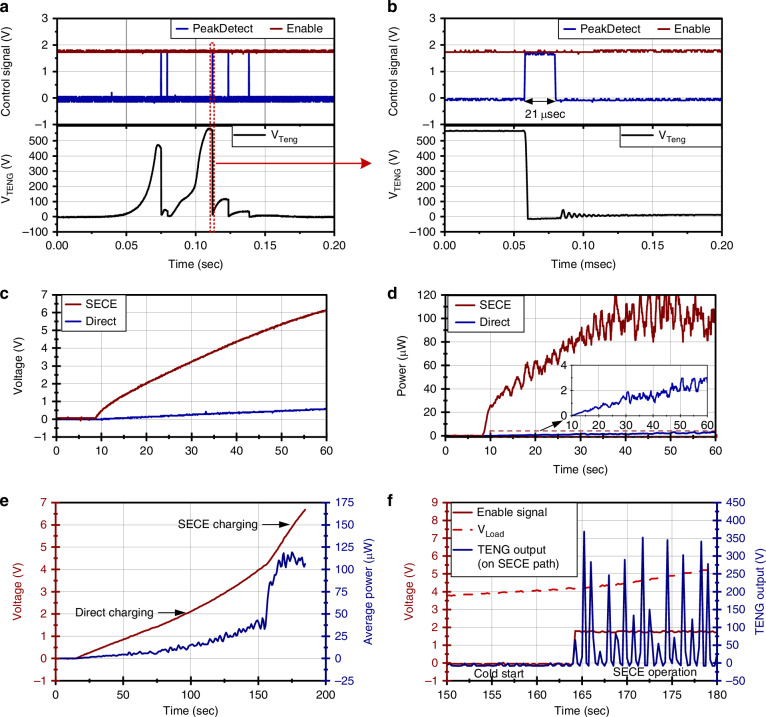
Characterization of the SECE circuit operation. **a** Measured waveforms of the SECE release for a single TENG cycle. **b** Zoomed-in view showing the energy transfer process. **c**, **d** Voltage and power when charging a 220 μF storage capacitor with and without SECE power management. **e** Charging a 100 μF storage capacitor from 0 V to operating voltage levels to demonstrate cold-start circuit operation. **f** Zoomed-in view of the transition from cold-start to SECE power management

The SECE power management circuit was implemented using discrete components on a PCB, and the previously characterized TENG operating at 5 Hz was connected to the power management circuit. Additional leakage paths from the power management circuit and temporal misalignment in the peak detection reduce the effective peak height of the TENG to 579 V and 463 V (Fig. 4a), compared with open-circuit voltage peaks of 723 V and 586 V. The circuit demonstrates successful execution of the SECE power management, where the voltage peak is detected, and the energy is released from the TENG. A zoomed-in view of the voltage drop shows that the TENG releases the energy for 21 μs, as controlled by a pulse from the monostable pulse generation circuit (Fig. 4b). Peak detector glitches are present but occur only after the primary SECE extraction, when most of the TENG energy has already been harvested, and therefore they result in negligible additional energy loss with minimal effect on overall power generation.

A comparison of power generation between the SECE circuit and the FBR circuit is performed with the power provided by a benchtop supply to drive the power management circuit (Fig. 4c, d). When charging a 220 μF capacitor, the conventional FBR circuit only reached 0.59 V with a charging time of 60 s, while the SECE circuit charged the capacitor to over 6.14 V. The SECE circuit also exhibits significantly higher instantaneous power compared to the conventional FBR circuit. At a voltage of 2 V, the SECE can generate an average of 57.95 μW, compared to 10.73 μW for the conventional FBR circuit, resulting in a 5-fold increase in power generation. When the storage capacitor is charged to a higher voltage, such as the 4.2 V needed for the SECE circuit operation to start, the power generated reaches an average of 108.43 μW with a maximum of 133.38 μW.

The operation of the cold-start circuit and the power management operation powered only by the TENG are shown in Fig. 4e, f. Initially, the storage capacitor is at 0 V and is charged directly by the TENG through the cold-start circuit. The initial power output is very low because the system initially uses the FBR circuit for charging. When using the FBR circuit, the power generated scales with voltage, and the TENG produces a roughly fixed charge per cycle, resulting in low power when voltage is low. As voltage increases, the instantaneous power provided by the TENG to the system increases gradually up to 38.29 μW at 4.2 V. Once the storage capacitor is charged to a sufficient voltage, the voltage monitor detects the voltage and produces an enable signal that enables the SECE circuit and turns off the direct charging route of the cold-start circuit. With the operation of the SECE circuit, the instantaneous power increases to an average of 110.78 μW, demonstrating a marked difference. The SECE circuit brings power generation to a level sufficient to continuously power microsystem operation.

### Fully integrated microsystem

A microsystem was created to demonstrate the applicability of the TENG and power management for powering sensing and continuous wireless communication by powering a microsystem that monitors VOC exposure in real time and continuously sends the data to a remote terminal using Bluetooth Low Energy (BLE). Exposure monitoring for VOCs is vital, as real-time detection of hazardous levels in occupational and urban environments enables accurate health-risk assessment and regulatory compliance^53–55^. While conventional methods rely on adsorbent tubes with delayed laboratory analysis, personalized, wearable options are needed to enable real-time detection, improving exposure assessment and workplace safety. Continuous monitoring is critical in this application, given the toxicity of VOCs, which range from respiratory irritation to cancer, particularly in settings with high chemical use and worker proximity to emission sources.

**Fig. 5:**
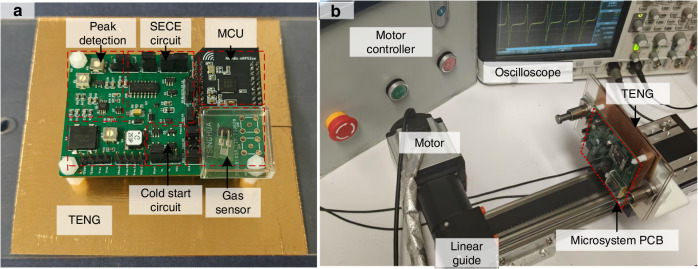
Self-powered microsystem for continuous sensing and wireless transmission. **a** Photograph of the assembled system, comprising the TENG, power management circuit, sensor, and MCU. **b** The test setup for the microsystem

The self-powered microsystem integrates a TENG, a SECE power management circuit with cold-start capability, a gas sensor, and an MCU, with an overall system size of 15 cm × 10 cm × 2 cm (Fig. 5a). The test setup consists of the microsystem, a motor and linear guide, a motor controller, an oscilloscope with high impedance probes to measure different voltages, and a laptop to receive the BLE transmissions (Fig. 5b). The TENG was operated using sliding motion provided by the linear guide and motor operating at 5 Hz. This frequency was selected because it falls within the range of human motion (1–5 Hz), corresponding to walking and light jogging, and is commonly adopted in wearable energy harvesting studies^18^. The storage capacitor for the microsystem was increased to 440 μF (using two 220 μF capacitors in parallel) in order to support MCU initialization and establish BLE operations without the storage capacitor voltage dropping below the operating voltage of the system. Individual components of the microsystem were first characterized independently, followed by full-system validation. The system operation was demonstrated starting from capacitor charging from 0 V via the TENG, transitioning to SECE power management, enabling MCU startup and BLE advertising, establishing a BLE connection, and achieving continuous sensing and wireless data transmission powered solely by the single low-frequency TENG.

#### Capacitive gas sensor design and characterization

**Fig. 6:**
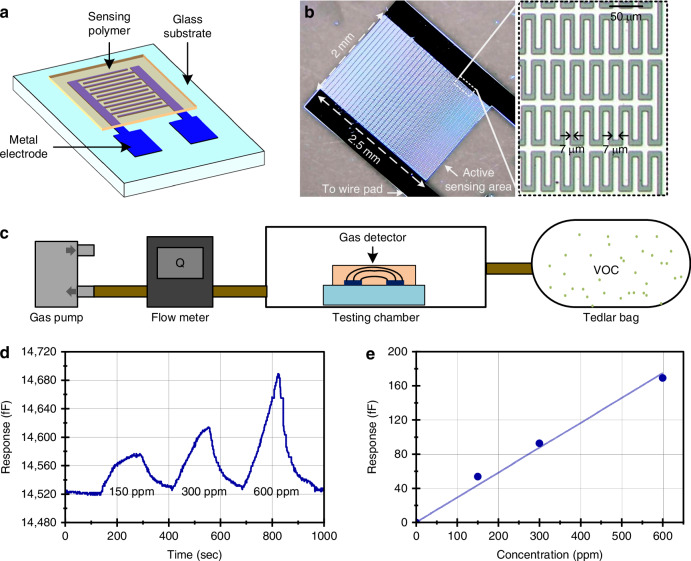
Capacitive gas sensor design and characterization. **a** Schematic of the capacitive gas sensor. **b** Photograph of the fabricated sensor and interdigitated electrodes. **c** Experimental setup for gas sensing. **d** Sensor response to varying concentrations of isopropyl alcohol. **e** Peak height at different concentrations, showing a proportional relationship

The gas sensor used in the microsystem is an interdigitated capacitive gas sensor with 7 μm electrode width and gap and an active area of 5 mm², coated with a thin PDMS layer that functions as the gas-sensitive film (Fig. 6a, b). The PDMS is spin-coated onto the substrate and has a measured thickness of 1.3 μm. When exposed to VOCs, the PDMS film undergoes changes in both the dielectric constant and thickness, leading to a measurable change in capacitance. Unlike traditional gas sensors that rely on chemical reactions or consumables, this design operates at room temperature, eliminating the need for heating elements and enabling low power consumption.

The gas sensor’s response to isopropyl alcohol (IPA) vapor, a characteristic VOC, was measured using a low-power capacitance-to-digital converter (CDC) with the test setup shown in Fig. 6c. The response of the gas sensor to IPA vapor with concentrations ranging from 100 ppm to 600 ppm is shown in Fig. 6d. The peak heights extracted from the plot varied proportionally with the corresponding vapor concentrations, showing a sensitivity of 0.282 fF/ppm, leading to a 3σ limit of detection of 9.12 ppm (Fig. 6e). The response time of the gas sensor is 93.8 s and the recovery time is 128.1 s. This response time is well-suited for low-power, long-term environmental or exposure monitoring applications where gas concentration typically varies over timescales of minutes rather than seconds, such as indoor air quality monitoring or industrial leakage detection. In such scenarios, the response time represents an acceptable trade-off for achieving fully self-powered operation under stringent energy constraints. The relatively quick response and recovery times for unpowered gas sensors are achieved due to the rapid diffusion and reversible adsorption of gas molecules within the thin, porous PDMS.

While the current sensor is sensitive to various gas species and subject to environmental influences such as humidity and temperature drift, these effects primarily manifest as baseline drift or slow variations relative to transient responses induced by chemical exposure. Such effects can be mitigated using low-complexity, computationally efficient algorithms for baseline correction and peak extraction (e.g., the continuous wavelet-based algorithm in ref. ^56^), which are well suited for the limited power budget of this microsystem. Furthermore, while the current unfunctionalized PDMS layer provides a broad response, chemical selectivity can be enhanced in future iterations through the application of targeted sensing polymers. For the purposes of this study, however, the sensor’s performance is sufficient to demonstrate the successful end-to-end operation of the energy-autonomous sensing architecture.

#### System integration and operation

**Fig. 7:**
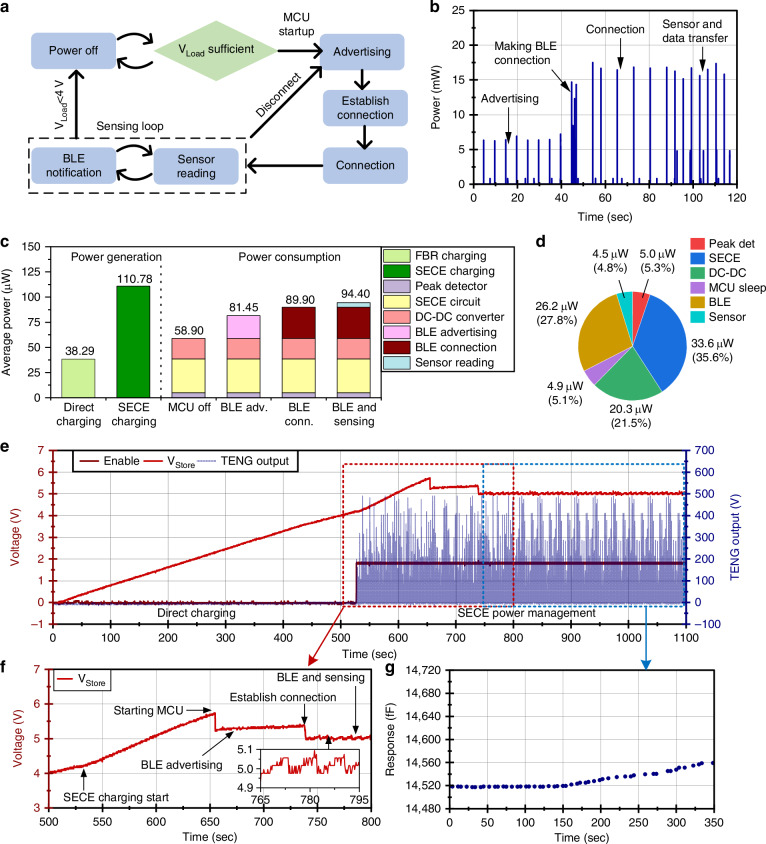
System operation of the microsystem. **a** State machine of the MCU operation. **b** Power consumption of BLE and sensor readout during operation. **c** Power generation and consumption breakdown of the system. **d** Breakdown of steady-state power consumption across system components, including MCU sleep, sensor readout, and BLE transmission. **e** Voltage reading of the storage capacitor during microsystem operation starting from cold-start operation. **f** Zoomed-in view of the *V*_*Store*_. **g** Sensor response to IPA headspace exposure with the microsystem self-powered by the TENG

As mentioned in the previous section, the microsystem consists of the TENG energy harvester, power management, gas sensor, readout circuit, and an MCU with BLE capabilities. The MCU control state machine is shown in Fig. 7a. When the storage capacitors are sufficiently charged, the MCU is powered on and begins BLE advertising. A laptop-based user interface detects the microsystem and attempts to establish a BLE connection. The establishment of the BLE connection requires a burst of high-frequency transmissions between the microsystem and the user interface to perform connection setup, service discovery, and security negotiations, which prevents immediate low-power operation. After this initial high power consumption, the MCU maintains a connection with the user interface for data transmission with much lower power consumption.

Through efficient power management and low-power system design, a balance between the harvested energy and consumed power is achieved, enabling continuous, real-time monitoring. The power consumption of the MCU and sensor during operation is shown in Fig. 7b. The MCU typically draws a baseline power consumption of 17.6 μW with short pulses of higher power consumption (mW level) associated with wireless BLE communication, processing, and sensor readout. The average power consumption during BLE advertising is 22.55 μW, increasing to 31.01 μW during BLE connection. The gas sensor itself consumes no power as it operates at ambient temperature, and the selected CDC chip is designed for low-power applications, consuming 4.50 μW on average with capacitance readings occurring every 5 s.

The power management circuit draws an average of 4.97 μW for the peak detection and 33.60 μW for the rest of the power management circuit when the TENG operates at 5 Hz. The DC-DC voltage converter is responsible for converting the harvested energy into a stable 1.8 V for the MCU and sensor operation. When converting 4.2 V to 1.8 V to provide power to microsystem electronics, the DC-DC voltage converter had an average power consumption of 20.33 μW.

A comparison between the power generated by the TENG and the power consumption of the system is shown in Fig. 7c. With the TENG operating at 5 Hz, a *V*_*OC*_ of 600 V, and the storage capacitor at 4.2 V, the power generated is 110.78 μW. The sensing and communication consume a combined 35.51 μW, while the power management circuitry consumes an additional 58.90 μW, resulting in a total system power consumption of 94.40 μW during operation. A breakdown of the steady-state power consumption across different operational states, including MCU sleep, sensor readout, and BLE transmission, is shown in Fig. 7d. The average power consumption of the power management circuitry is comparable to or higher than that of the MCU, sensor, and BLE because the latter are duty-cycled and remain in low-power sleep modes for most of the operating cycle (averaging 4.5 μW), whereas the power management circuits must operate continuously to monitor the TENG output and regulate energy transfer. Such a trade-off is typical in self-powered systems, where the always-on energy harvesting interface can dominate the power budget, highlighting the importance of efficient power management design. Overall, the total system power consumption remains below the harvested power delivered to the storage capacitor, demonstrating the feasibility of sustained operation for continuous sensing and wireless communication using harvested energy. Detailed plots of the power consumption measurement can be found in Section S5 of the Supporting Information.

The complete operation of the microsystem was validated by monitoring the storage capacitor voltage (*V*_*store*_) during a representative harvesting and sensing cycle, as shown in Fig. 7e. Starting from 0 V, the capacitor was initially charged through the cold-start circuit until the enable threshold for the SECE power management was reached, corresponding to the initial voltage rise in the plot from 0 s to 525 s. Once sufficient energy was accumulated, the routing of the TENG output into the SECE power management, and the steeper rise in the voltage charging profile was observed. After a 125 s charging period using the SECE power management, the storage capacitor reached 5.6 V, and the MCU was manually powered on to begin BLE advertising. This startup process draws significant power, reducing the stored energy and dropping the capacitor voltage from 5.6 V to 5.2 V (corresponding to an energy consumption of 847.1 µJ) during a duration of 50 ms. During advertising, the power consumption of the system is sufficiently low that the storage capacitor continues to charge slightly, resulting in a voltage increase from 5.2 V to 5.4 V over 80 s. A BLE connection is then initialized from the GUI. The initial ~2 s period of the BLE connection is power-intensive due to the high power consumption required for connection setup, service discovery, and security negotiations. This connection process uses a total of 496.4 µJ over a period of 2 s, causing a noticeable drop in the *V*_*store*_. Following the establishment of the BLE connection, the MCU performs readout of the gas sensor and transmits the data over BLE, with each transmission event producing observable dips in the *V*_*store*_ (Fig. 7f). With each BLE transmission, the voltage drops to 5.0 V before being charged to 5.1 V for the next BLE message. Continuous operation of the self-powered microsystem was demonstrated, during which an IPA exposure event was successfully recorded and transmitted continuously (Fig. 7g). This data was sent to the GUI and is plotted. These results demonstrate the platform’s capability for continuous, real-time VOC monitoring in ambient air, enabling early warnings for harmful VOC exposure.

### Performance comparison and outlook

Compared to prior self-powered systems that relied on hybrid energy harvesters, multiple TENGs, high-frequency mechanical energy, or achieved only intermittent wireless transmission after long charging times (Table 1), the presented work demonstrates continuous wireless operation powered by a single TENG device, representing an important step toward fully self-powered microsystems. This improvement is enabled by the co-design of the energy harvester, power management circuitry, and low-power sensing and communication electronics, which together enable continuous operation under low-frequency mechanical excitation.

**Table 1:** Comparison of self-powered microsystems for continuous wireless sensing and communication

Energy Source	# of TENGs	Hybrid energy harvesting?	TENG working mode	Charging period / Continuous operation	Communication protocol	Ref.
Human motion	6	No	Sliding	60 sec	Bluetooth	^27^
Engine vibration	7	No	Contact-separation	Continuous	Bluetooth	^28^
Human motion	1	Yes	Contact separation	Continuous	Bluetooth	^29^
Airflow	2	No	Contact-separation	98 sec	RF transmission	^57^
Airflow	2	No	Contact-separation	40 min	Bluetooth	^58^
Human motion	8	No	Sliding	45 min	Bluetooth	^59^
Human motion	1	No	Contact-separation	Continuous	Bluetooth	This work

This microsystem offers significant potential for further optimization. While the system demonstrates self-powered operation, environmental variations such as humidity, temperature, airborne interferents, and fluctuating mechanical energy could influence sensing accuracy and power stability. Similarly, scaling to multi-node networks and high-volume manufacturing will require optimization of packaging, integration, and long-term durability. These challenges represent important directions for future work, focusing on environmental robustness, real-world validation, and scalable manufacturing.

In addition, it should be noted that the current system is characterized under controlled periodic excitation, which does not fully capture the intermittent nature of real human motion. Therefore, the present work serves as a proof-of-concept of the viability of continuous self-powered operation under controlled yet representative low-frequency human-motion conditions. Future work will focus on experimental validation under realistic human-motion profiles, including non-periodic and activity-dependent excitations, thereby enabling more comprehensive and application-relevant real-world testing.

Further improvements are possible through tighter electronics integration. Specifically, transitioning from discrete components to a custom CMOS implementation of the SECE power management and cold-start circuitry could significantly reduce both power consumption and overall footprint. Incorporating on-chip micro-supercapacitors as energy storage could further reduce system size and enable fully monolithic integration. In parallel, future system-level packaging will incorporate specialized insulation and encapsulation strategies to maintain reliable operation and ensure the safe handling of high-voltage outputs. While the TENG’s high open-circuit voltage presents minimal safety risks due to its inherently low current output, robust encapsulation remains essential for practical deployment to shield the device from ambient humidity and environmental contaminants. These changes, together with targeted advances in TENG design, would allow the TENG to be scaled down, enabling a compact system that integrates a smaller TENG with the IC while maintaining sufficient energy output for continuous operation. Such advances would not only further improve efficiency but also enhance the portability, scalability, and applicability of TENG-powered microsystems, enabling broader practical deployment in environmental monitoring, IoT sensing, and autonomous microsystem platforms.

## Conclusion

This work demonstrates a self-powered microsystem capable of continuous wireless sensing and data transmission using only a single TENG operating at low frequency as its sole energy source. By utilizing a high-voltage–tolerant power management circuit and low-power sensing and communication electronics, the system effectively mitigates the mismatch between the pulsed, high-voltage output of TENGs and the low-voltage requirements of the microsystem electronics, increasing the usable harvested power by a factor of five. Under 5 Hz mechanical excitation, the microsystem is able to start from a fully discharged state and achieves continuous wireless operation with a total power consumption of 94.40 μW, which is lower than the harvested power of 110.78 μW. Unlike prior TENG-powered systems that rely on hybrid harvesters, multiple devices, or intermittent transmission after extended charging periods, this platform demonstrates sustained, self-powered operation using a single harvester under practical conditions. Overall, this work highlights the importance of system-level integration and optimization across energy harvesting, power management, sensing, and communication in microsystems. By addressing these elements jointly rather than in isolation, TENG-powered microsystems can transition from laboratory demonstrations toward scalable, maintenance-free microsystems platforms for sustainable autonomous sensing in IoT applications.

## Materials and Methods

### TENG energy harvester

The TENG energy harvester operated in contact-separation mode. Acrylic with a thickness of 2 mm was cut into 15 × 10 cm. A polytetrafluoroethylene (PTFE) film and polyimide with a thickness of 0.05 mm served as the negative and positive triboelectric materials, with copper foil used as the electrode. The effective contact area was 10 × 10 cm with holes laser cut on the outside sides of the active area. Linear Bearings are placed in the holes to guide the contact-separation movement in order to provide a perfectly vertical and parallel separation movement.

### Electronics and system fabrication

The electronics for the system were designed using JLC EDA and were fabricated by a commercial manufacturer. The details of the circuit schematics, layout, and bill of materials are described in Section S4 of the Supporting Information. The MCU module (nRF52832, Nordic Semiconductor, Oslo, Norway) was programmed using Visual Studio Code. The BLE communication used an advertising interval of 5 s and a connection interval of 3 s. The gas sensor readings, performed by the MCU every 5 s, are enabled by a low-power capacitance-to-digital converter (AD7158, Analog Devices, Inc., Wilmington, MA, USA) that ensures precise measurements with minimal power use. The GUI used to receive the sensor data was programmed in Python and is responsible for scanning for BLE advertising, establishing the BLE connection, reading and parsing the sensor data, plotting it in real time, and allowing the data to be saved for further analysis. The details of the GUI are described in Section S6 of the Supporting Information.

### Capacitive gas sensor

The gas sensor was fabricated through deposition of metal onto a glass substrate before coating with a sensing polymer. Starting with a 400 μm thick glass substrate, lithography and evaporation were used to deposit 20/100 nm of Ti/Pt to form the interdigitated electrodes. The electrodes are then coated with a 1–4 μm thick layer of PDMS (Sylgard 184, Dow Inc., MI, USA) through spin coating with different spin speeds, determining the coating thickness. Curing of the PDMS was performed at 150 °C for 1 hour. Different concentrations of IPA vapor were prepared in a Tedlar bag by injecting precise volumes of liquid IPA, allowing complete evaporation, and diluting with zero air to achieve the desired ppm levels. A diaphragm gas pump (KLVP08, Kamoer Fluid Technology Co., Ltd., Shanghai, China) is used to draw the vapor into a test chamber with the gas sensor at a set flow rate of 10 sccm to introduce either the vapor mixture or ambient air for testing.

## Supplementary information

Supporting Information
