# Supplementary material for: A self-powered microsystem with efficient power management for continuous wireless sensing
Source: Microsyst Nanoeng. 2026 May 13;12:178. doi: 10.1038/s41378-026-01315-z (PMC13172475; doi:10.1038/s41378-026-01315-z)
Supplement: Supplementary file 1 — Supporting Information [file 41378_2026_1315_MOESM1_ESM.pdf]

Supporting Information

A Self-Powered Microsystem with Efficient Power Management for Continuous Wireless Sensing

Xiangyu Zhao[1], Zerui Xu[1], Ziyang Ou[1], Qingfeng Wu[1], Yifeng Liu[1], Yuqi Kang[1], Philippe Basset[2], and Xiaohong Wang[1]

[1] School of Integrated Circuits, Tsinghua University, Beijing 100084, China

[2] Univ Gustave Eiffel, CNRS, ESYCOM, F-77454 Marne-la-Vallée, France

*Corresponding author

Email: X.W. (wxh-ime@tsinghua.edu.cn)

S1. Operating Principle of TENG
S2. Comparison of TENG Power Management
S3. Detailed Power Management Design
S4. Circuit Schematic and Layout
S5. Power Consumption Plots
S6. Graphical User Interface
S7. Oscilloscope Figures

S1. Operating Principle of TENG

The triboelectric effect involves generating electrical charges when two materials with different electron affinities touch and then separate. This process results from contact electrification and electrostatic induction, which causes a net transfer of charge between the surfaces. When they are separated, an electric potential difference forms, enabling electrons to flow through an external circuit to balance the electrostatic field. Known for causing everyday static electricity, the triboelectric effect has been used at the nanoscale to develop triboelectric nanogenerators (TENGs), which convert ambient mechanical energy into electrical power.

TENGs typically operate in four configurations: contact-separation, lateral-sliding, single-electrode, and freestanding triboelectric-layer modes. Among these, the contact-separation mode is the most common in the literature (Fig. S1a). In this mode, two dielectric layers with different triboelectric polarities repeatedly contact and separate under external mechanical stimuli. During contact, charge transfer takes place at the interface; when separated, an electrostatic potential difference causes electrons to flow between the back electrodes through an external circuit, producing alternating current that can be stored or used to power electronics. The voltage and current outputs depend on the surface charge density (σ), the dielectric thickness (d), and the separation distance (x). The equation for voltage and capacitance of TENG is given as:

$$V_{OC} = \frac{\sigma x(t)}{\varepsilon_0} \tag{S10}$$

$$C_{TENG} = \frac{\varepsilon_0 A}{x(t) + \frac{d}{\varepsilon_d}} \tag{S11}$$

where ε_0 is the vacuum permittivity, ε_d is the permittivity of the dielectric, and A is the effective contact area. It can be seen from these two are dependent on the distance of the gap and the maximum voltage occurs when the plates are at their furthest distance. The output of a TENG operating in contact-separation mode has positive peak, followed by a negative peak, corresponding to the pressing and releasing motions, respectively. (Fig. S1b)

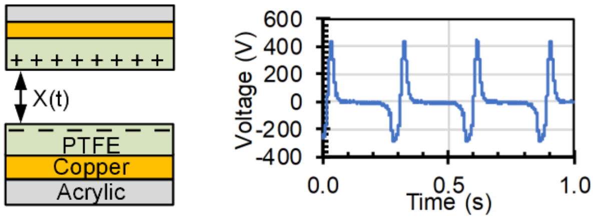

(a) (b)

Fig. S1: TENG operating in contact separation mode. (a) The general structure and (b) the output of the TENG.

The other TENG modes extend the same basic principle to different mechanical setups. In the lateral-sliding mode, horizontal relative motion replaces vertical separation, producing periodic charge transfer through sliding friction. The single-electrode mode removes the need for two fixed electrodes by referencing one to ground, enabling use in freely moving or wearable systems. Lastly, the freestanding triboelectric-layer mode uses a mobile charged dielectric between stationary electrodes, allowing charge induction without direct contact with electrodes. Together, these modes enable TENGs to efficiently harvest various types of low-frequency mechanical energy, making them a versatile platform for providing power for self-powered microsystems.

S2. Comparison of TENG Power Management

Typical methods for power management for TENG output to convert the inherently alternating signal into usable direct current for electronic loads include the full-bridge rectifier (FBR), the synchronized switch harvesting on inductor (SSHI) method, and the synchronous electric charge extraction (SECE) strategy. Among these, the FBR is the simplest and most conventional approach, rectifying the alternating output through diodes to produce DC voltage; however, its energy conversion efficiency is limited by voltage drops and impedance mismatch between the TENG and the load. The SSHI method improves energy transfer by momentarily connecting an inductor across the TENG at each displacement extremum, inverting the voltage across the device to enhance charge flow and thereby increase the harvested power. In contrast, the SECE technique extracts the accumulated charge synchronously at the voltage peak via a controlled switch and inductor, allowing nearly all the electrostatic energy stored in the TENG's capacitance to be transferred to the load.

To compare the three methods, the theoretical efficiency of extracted energy is compared. To calculate the efficiency, the concept of cycles of maximum energy output (CMEO) and the efficiency is the output per-cycle (E_{Extr}) divided by the maximum energy output per-cycle (E_{CMEO}), which can be calculated using the following equations:

$$E_{Extr} = \int_0^T V_T I_T \, dt = \int_0^T V_T \, dQ_{C_T} \tag{S1}$$

$$E_{CMEO} = \frac{1}{2} Q_{SC,MAX}\left(V_{OC,MAX}^{p} + V_{OC,MAX}^{n}\right) \tag{S2}$$

where $Q_{SC,MAX}$, $V_{OC,MAX}^{p}$, $V_{OC,MAX}^{n}$ are the minimum short-circuit charge, positive maximum open circuit voltage, and negative maximum open circuit voltage of the TENG. Meanwhile, $Q_{SC,MAX}$ can be calculated as:

$$Q_{SC,MAX} = V_{OC,MAX}^{p} C_{min} = V_{OC,MAX}^{n} C_{max} \tag{S3}$$

where C_{min} and C_{max} are the minimum internal capacitance and the maximum internal capacitance of the TENG at the maximum displacement and minimum displacement, respectively. β is defined as the ratio of C_{max} to C_{min}, and is expressed as

$$\beta = \frac{C_{max}}{C_{min}} = \frac{V_{OC,MAX}^{p}}{V_{OC,MAX}^{n}} \tag{S4}$$

Therefore, Equation (S4) can be expressed as:

$$E_{CMEO} = \frac{1}{2}\left(1 + \frac{1}{\beta}\right)\left(V_{OC,MAX}^{p}\right)^{2} C_{min} \tag{S5}$$

We can define the extraction efficiency η_{Extr} by dividing the energy transferred to the load per-cycle by the E_{CMEO}:

$$\eta_{Extr} = \frac{E_{Extr}}{E_{CMEO}} \times 100\% \tag{S6}$$

For the FBR method, the energy extracted (E_{FBR}) is:

$$E_{FBR} = V_R Q_{Extr} = 2 V_R C_{min}\left[V_{oc,max} - (1 + \beta) V_R\right] \tag{S7}$$

Where V_R is the load voltage. When $V_R = \frac{V_{oc,max}}{2(1+\beta)}$, E_{FBR} reaches its the maximum value of $E_{FBR,max} = \frac{C_{min} V_{oc,max}^{2}}{2(1+\beta)}$.

For the SSHI method, the energy that can be extracted per-cycle is given by:

$$E_{SSHI} = V_R\left(\left|\Delta Q_{CT}^{p}\right| + \left|\Delta Q_{CT}^{n}\right|\right) = 2 V_R C_{min} V_{oc,max} \tag{S8}$$

Similarly, the extracted energy of the SSHI is still affected by the load voltage V_R. When it comes

to TENG, the open-circuit voltage is hundreds time higher than V_R, resulting in significant efficiency attenuation.

For the SECE method, the energy that can be extracted per-cycle is given by:

$$E_{SECE} = \frac{1}{2}\left(1 + \frac{1}{\beta}\right) C_{min} V_{oc,max}^2 \quad \text{(S9)}$$

The energy extracted is no longer dependent on the load voltage, and instead is dependent on the V_{oc} of the TENG and can theoretically reach the maximum energy output per-cycle (E_{CMEO}). The E_{Extr} and η_{Extr} for FBR, SSHI, and SECE technique are shown in Table S1.

Table S1. Comparison of the maximum energy extracted by different power management methods

	E_{Extr}	Theoretical η_{Extr}	Dependent on V_R
**FBR**	$2V_R C_{min}\left[V_{oc,max} - (1+\beta)V_R\right]$	$\frac{4\left[\beta V_{oc,max} - \beta(1+\beta)V_R\right]}{(1+\beta){V_{oc,max}}^2} \leq 25\%$	Yes
**SSHI**	$2V_R C_{min} V_{oc,max}$	$\frac{4\beta V_R}{(1+\beta)V_{oc,max}} \leq 100\%$	Yes
**SECE**	$\frac{1}{2}\left(1+\frac{1}{\beta}\right) C_{min} V_{oc,max}^2$	$\approx 100\%$	No

S3. Detailed Power Management Design

The TENG harvests low-frequency vibrational energy and generates a high-voltage, pulsed output. This output is rectified with a full-wave diode bridge and fed into a peak detector circuit. The peak detector circuit detects peaks in the TENG output by detecting zero crossings in its temporal derivative and is composed of a differentiator followed by a comparator with hysteresis. The use of the peak detection circuit allows for a wide range of low-frequency or even aperiodic vibration movement. As the peak voltage plays a significant role in the energy generated, precise peak detection to determine the optimal moment for energy extraction is essential, and the effects of nonidealities such as RC delays, parasitic effects, and noise need to be mitigated. This peak detection circuit is optimized by adjusting the V_{ref} of the comparator to counteract the RC delay introduced in the differentiator portion and the thresholds of the comparator for false-trigger prevention.

The detected peak signal is then fed into a monostable pulse generation circuit. The monostable pulse generation circuit is normally low and a pulse is generated when a peak is detected to signal the SECE to transfer the TENG's energy. The pulse width needs to be sufficiently long so that complete energy transfer can be achieved. The release process should end when the voltage of the TENG returns to nearly zero, denoting complete energy transfer. A comparator is used to determine when the TENG voltage has decreased sufficiently, and due to the high voltage of the TENG, a GΩ voltage divider is used to divide the TENG voltage by 100 while minimizing the energy loss before being fed into the comparator to allow the high voltage of the TENG to interface with the conventional circuits.

The flyback voltage converter circuit is composed of a field effect transistor and a coupled inductor, specifically selected to handle the high output voltage of the TENG. A GaN HEMT with a drain to source voltage rating of 600 V was selected to handle the high voltage output of the TENG, with its low ON resistance and high

switching speed to minimize power dissipated. The flyback circuit efficiently transfers energy from the TENG by applying the TENG’s high-voltage, pulsed output on the primary side of the transformer when the switch is closed, and then releasing the charge through a diode into a storage capacitor on the secondary side when the switch opens. It handles TENG's high voltage, isolates the output, adapts to aperiodic pulses, and addresses the impedance mismatch between the storage element and TENG while stepping down the voltage for practical storage in a storage capacitor and use to power conventional electronics that comprise IoT microsystems.

The SECE electronics and control signals are designed to operate at 1.8 V to reduce power consumption and to match the voltages required for the rest of the microsystem. The only exception is a level shifter that is used to interface between the monostable pulse generation and the flyback circuit so that the voltage of the signal can be changed to control the high voltage MOSFET in the flyback circuit.

While SECE power management is more efficient, an initial voltage is needed to power the components of the SECE circuit. A cold start circuit is implemented to provide a charging path for the system to initialize when the system is in a completely discharged state. The cold start circuit is composed of a voltage monitor connected to a depletion-mode PMOS. When the voltage in the storage capacitor is insufficient for SECE power management operation, the storage capacitor is directly charged by the TENG. Once sufficient energy is harvested, a voltage monitor chip detects the voltage and sends an enable signal to activate the buck converter, which powers the SECE power management circuitry, and disables the direct path between the TENG and the storage capacitor. The buck converter converts the energy stored in the storage capacitors into a stable 1.8 V to power the SECE power management circuitry and the subsequent sensor, readout circuit, and microcontroller. The specifics of the electronics can be seen in Section S6.

S4. Circuit Schematic and Layout

The circuit for the system can be split into the peak detection circuit (for detecting the peaks in the rectified TENG output to enable the SECE power management), the SECE power management circuit (to efficiently convert the energy from the TENG), the DC/DC converter (for providing a stable voltage to power the MCU and some parts of the SECE), sensor readout (to read out the gas sensor), and the MCU module (to control the sensor readout and BLE communications). The circuit schematic can be seen in Fig. S2, the layout in Fig. S3, and the component list in Table S2.

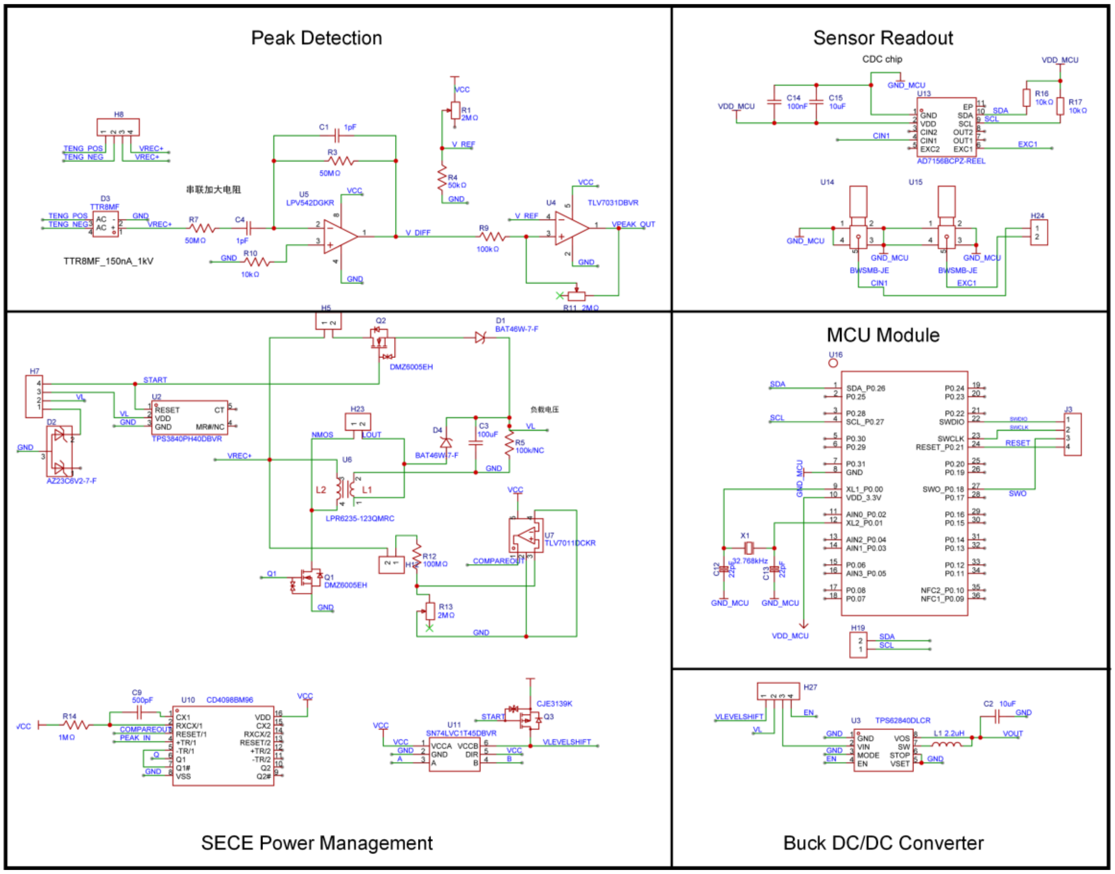

Fig. S3: Circuit schematic of the peak detection, power management, DC/DC converter, sensor readout, and BLE modules.

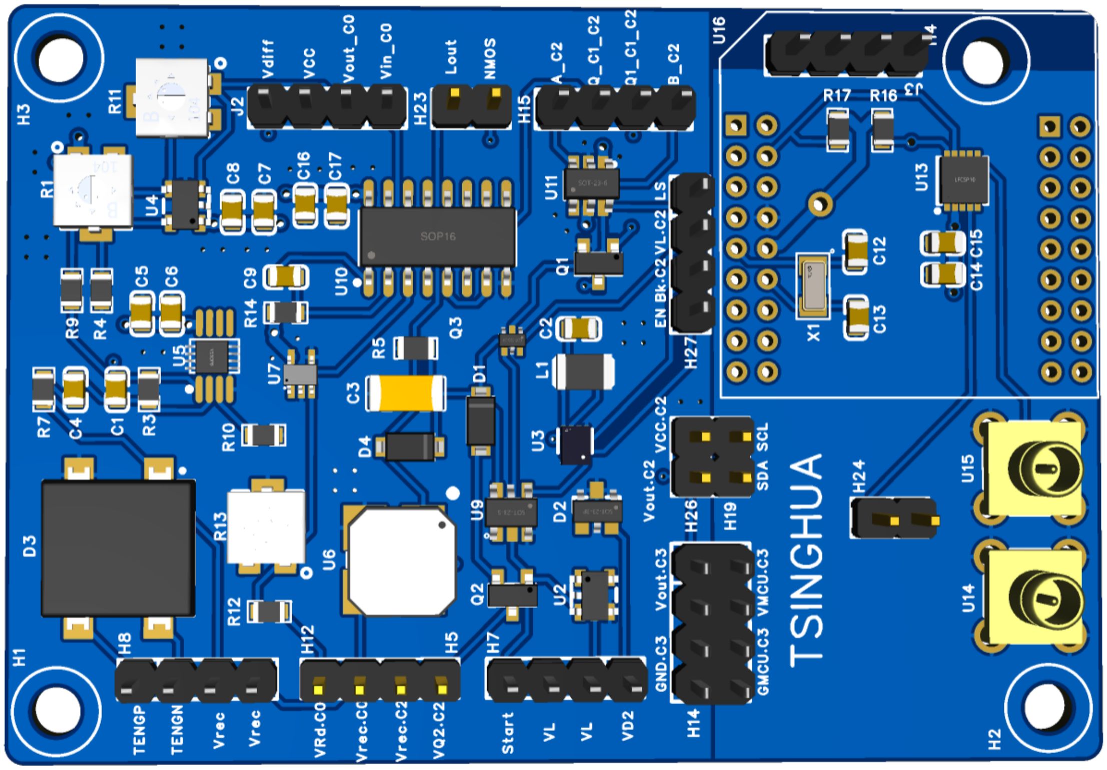

Fig. S4: Layout of the microsystem circuits.

Table S2. Key components and their specifications used in the SECE power management circuit and microsystem implementation to facilitate reproducibility.

Designator	Value/Series Number	Description	Key Parameter
C1,C4	1pF	0603 Capacitor	
C2,C6,C8,C15,C17	10uF	0603 Capacitor	
C3	100uF	Storage Capacitor	
C5,C7,C14,C16	100nF	0603 Capacitor	
C9	500pF	0603 Capacitor	
C12,C13	22pF	0603 Capacitor	
D1,D4	BAT46W-7-F	High speed rectification	Vf ≈ 450 mV, low leakage
D2	AZ23C6V2-7-F	Zener diodes	Vz ≈ 6.2 V
D3	TTR8MF	Switching diode	
L1	FCI201209F-2R2K	0805 Inductor	2.2 μH, DCR = 0.65Ω, Isat = 30 mA
Q1	CMN3160M	GaN HEMT	600V, Rds(on) = 45Ω
Q2	DMZ6005EH	Depletion mode MOSFET	
Q3	CJE3139K	N-Channel JFET	
R1,R11,R13	2MΩ	Potentiometer	
R3,R7	50MΩ	0603 Resistor	
R4	50kΩ	0603 Resistor	
R9	100kΩ	0603 Resistor	
R10,R16,R17	10kΩ	0603 Resistor	
R12	100MΩ	0603 Resistor	
R14	1MΩ	0603 Resistor	
U2	TPS3840PH40DBVR	Voltage Supervisor	4.0 V threshold, ultra-low IQ
U3	TPS62840DLCR	Buck DC/DC Converter	
U4	TLV7031DBVR	Comparator	Low IQ
U5	LPV542DGKR	Op Amp	Low-power
U6	LPR6235-123QMRC	Op Amp	
U7	TLV7011DCKR	Comparator	
U9	SN74AHC1G14DBVR	Schmitt Trigger	
U10	CD4098BM96	Monostable Multivibrator	
U11	SN74LVC1T45DBVR	Level Shifter	
U13	AD7156BCPZ-REEL	Capacitance Readout	24 bit high resolution
U16	nrf52832 Module	BLE MCU module	
X1	32.768kHz	Quartz Crystal Oscillator	

S5. Power Consumption of the Microsystem

The power consumption of the microsystem was measured using the Nordic Power Profiler Kit II. When the TENG is operating at 5 Hz, the peak detector has an average current draw of 2.76 μA which corresponds to 4.97 μW as it operates at 1.8 V. The total power consumption of the SECE circuit is 8 μA when operating at 4.2 V powered by a benchtop supply. When using the DC-DC converter to power the circuit an average current draw of 12.84 μA is observed, meaning that the DC-DC converter has an overhead of 20.3 μW (Fig. S4a). Another significant source of the main current draw is from the MCU responsible for performing the sensor readout and data transmission (Fig. S4b). The power consumption of advertising, connection, and sensor and data transfer were averaged over a 10 second time period. While the deep sleep power consumption is very low, sending advertising or connection packets has short pulses of very high power consumption. The current consumption of single BLE advertising and connection messages can be seen in Fig. S4c and Fig. S4d, respectively. The average power consumption for advertising, connection, and connection with sensor operation to be 22.553 μW, 31.008 μW, 35.51 μW respectively. Another period of high power consumption is the initial MCU startup which has a large spike of power of over 100 mW using 169.41 μC of charge at 5V, corresponding to 847.05 μJ of energy (Fig. S4e). Establishing BLE connection requires a few seconds of high frequency communication between the which draws a total of 496.44 μJ (Fig. S4f).

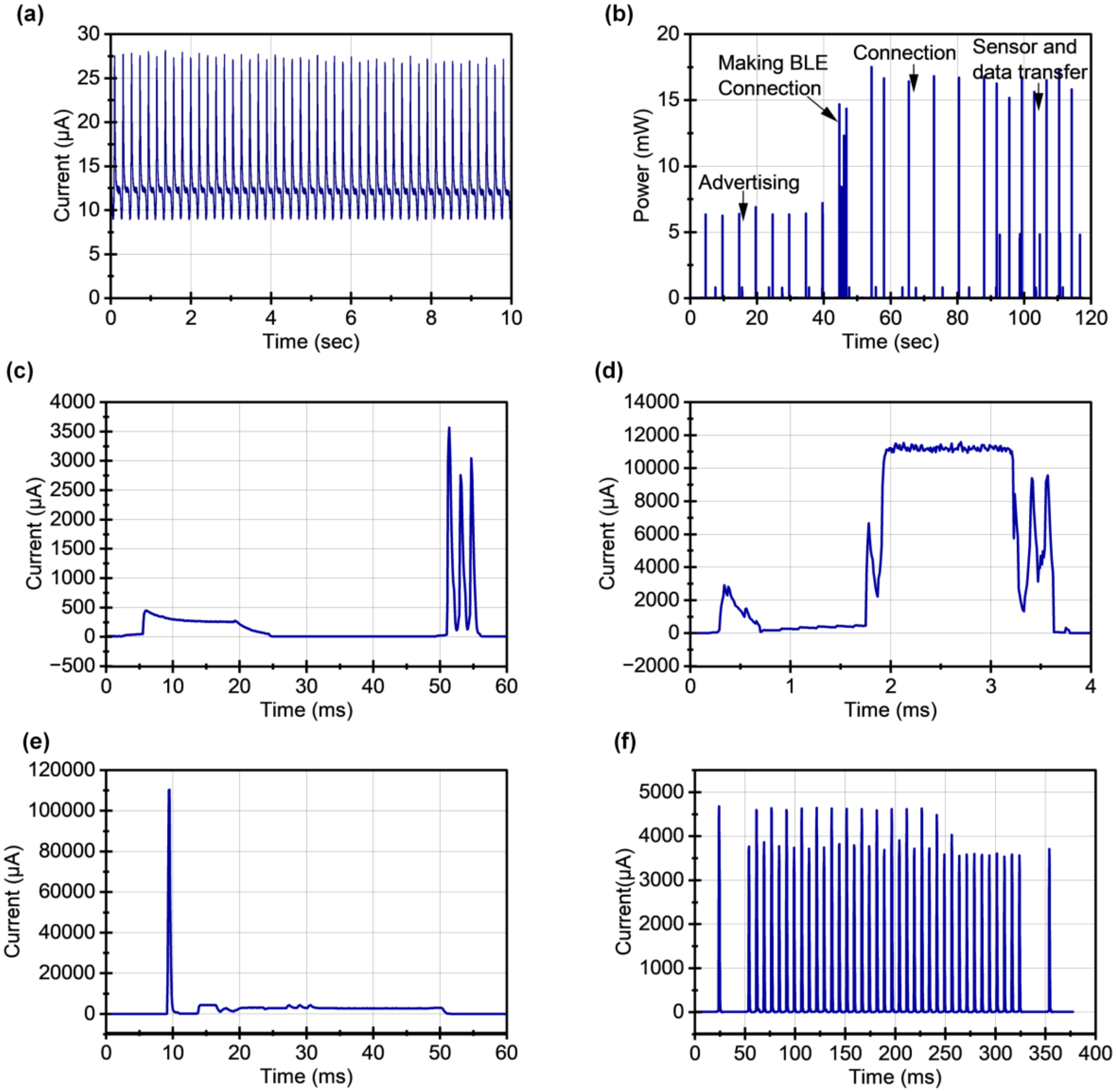

Fig. S4: Power consumption of the microsystem. (a) The total current draw (at 4.2 V) of the SECE power management circuit including the DC-DC converter during 5 Hz TENG operation. (b) The power consumption during different parts of the MCU operation. (c) An individual BLE advertising event. (d) An individual BLE connection event. (e) Power consumption of MCU initialization. (f) Power consumption of establishing the BLE connection.

S6. Graphical User Interface

A custom Python graphical user interface (GUI) developed with the Tkinter framework is made to acquire, process, and visualize data from a gas sensor in real-time (Fig. S5). The software initializes a Bluetooth Low Energy (BLE) scan to discover and connect to a dedicated microsystems platform, with the requisite BLE service and characteristic identifiers hardcoded into the microcontroller firmware. Upon a successful connection, the application enables notifications for the designated data stream. The data in the notifications sent by the microsystem, structured as 2-byte little-endian integers representing raw readings from a Capacitive-to-Digital Converter, are parsed and converted into capacitance in fF. These capacitance values are then appended to a real-time plot. This plot provides a real-time visual of the measurements while also allowing the data to be saved for further analysis at a later time.

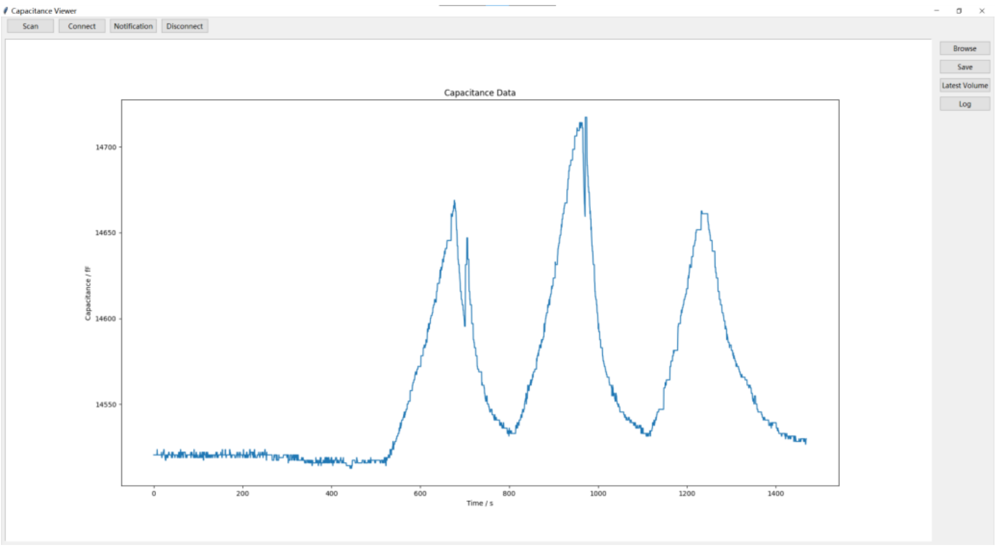

Fig. S5: GUI responsible for parsing the results and providing a real time display.

S7. Oscilloscope Figures

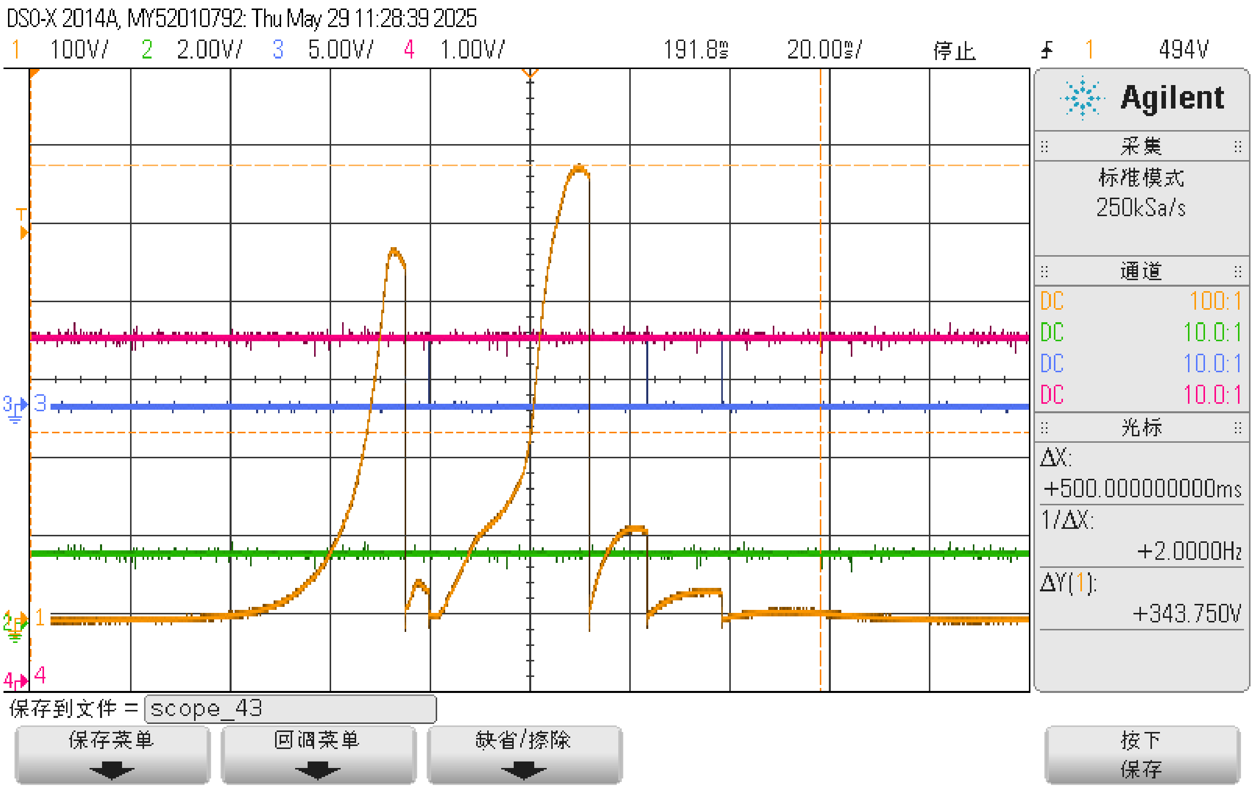

Fig. S6: Waveforms of the SECE circuit operating to transfer the power generated by the TENG. The yellow trace is the TENG voltage, the green trace is the enable signal, the blue trace is the peak detection circuit output, and the pink trace is the voltage of the storage capacitor.

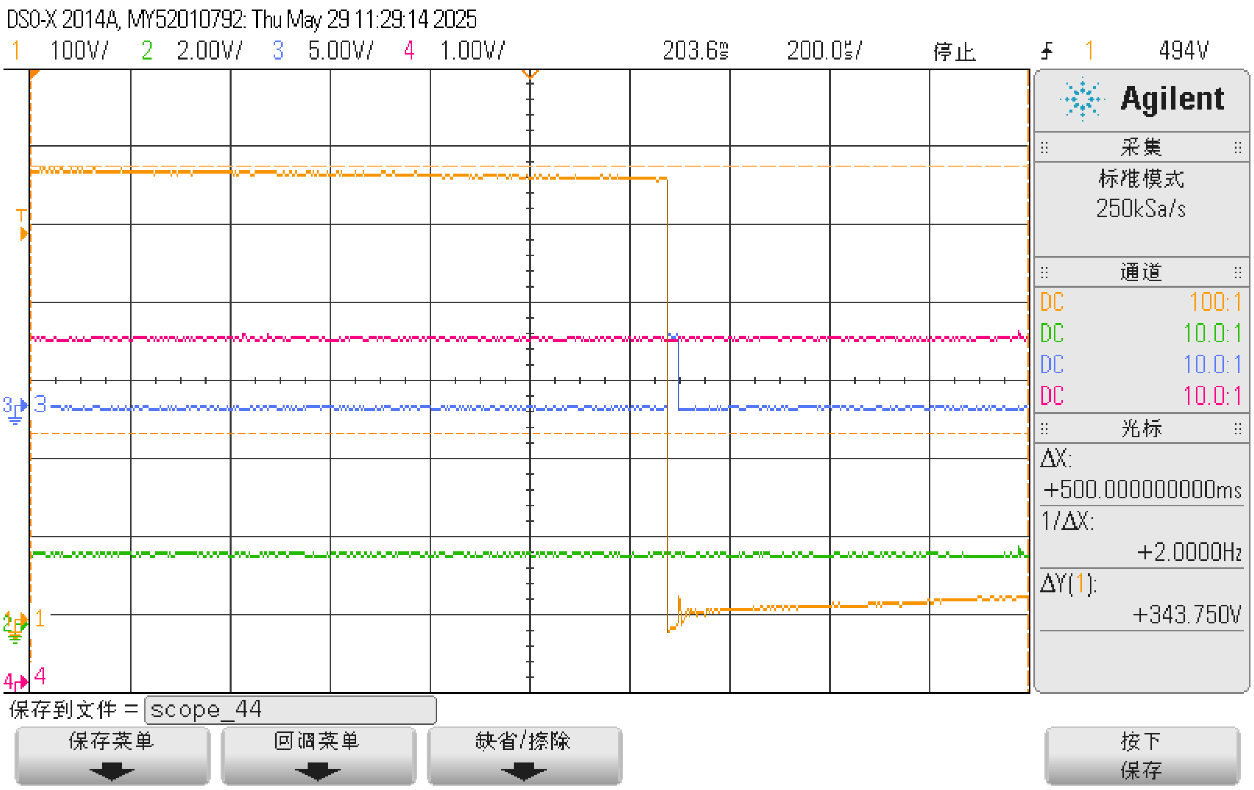

Fig. S7: Zoomed-in waveform of the voltage drop initiated by the peak detection circuit. The yellow trace is the TENG voltage, the green trace is the enable signal, the blue trace is the peak detection circuit output, and the pink trace is the voltage of the storage capacitor.

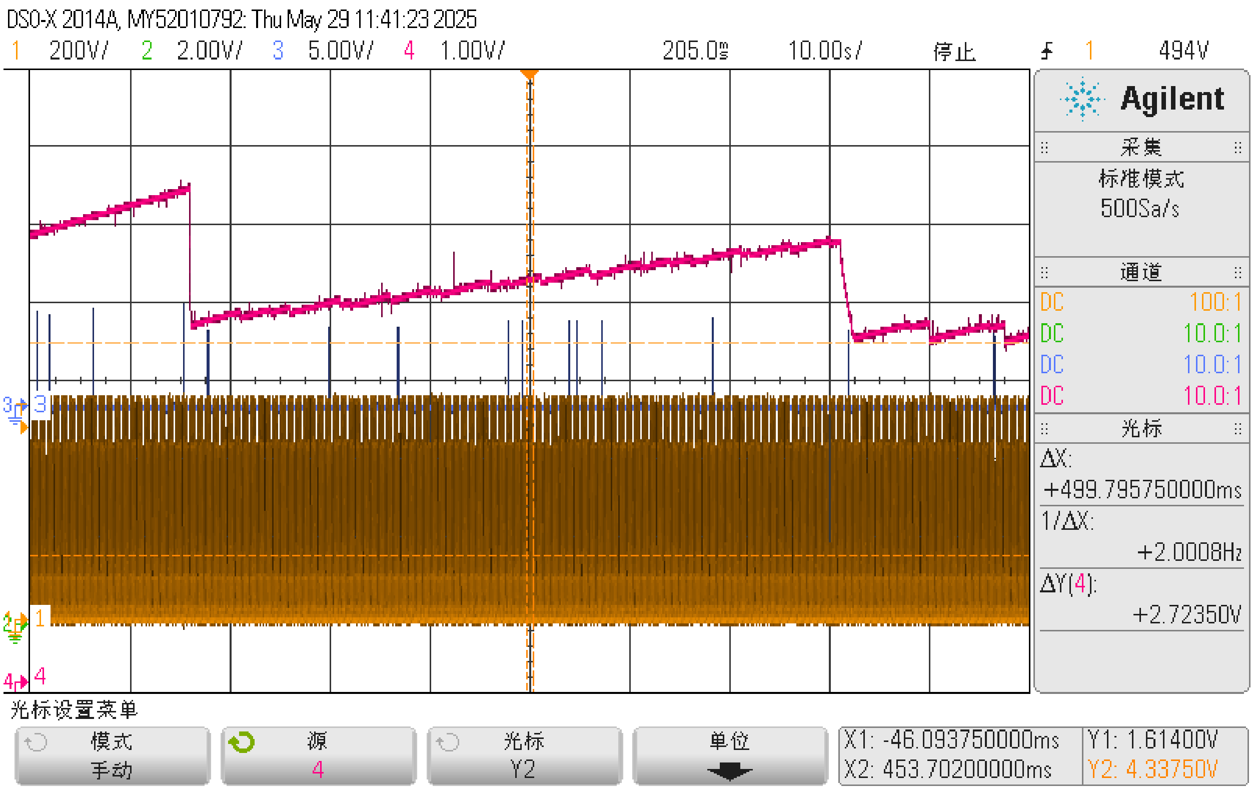

Fig. S8: Voltage change in the storage capacitor during microsystem operation. Voltage stored in the capacitor sees a drop as the MCU is started. The voltage recovers slightly during the course of BLE advertising. Establishing the BLE connection sees a second large drop in voltage. and periodic sensing and data transfer. After the connection is established, the power generated is sufficient to continuously drive data collection and wireless communication. The yellow trace is the TENG voltage, and the pink trace is the voltage of the energy storage capacitor.
